# Blood Pressure Estimation Through Pulse Wave Analysis Using Features Extracted from Carotid Diameter Distension Waveforms

**DOI:** 10.3390/bios16030151

**Published:** 2026-03-08

**Authors:** Lirui Xu, Zhenhua Li, Pan Xia, Chen Zhang, Lidong Du, Wei Tian, Zhen Fang

**Affiliations:** 1 Naval University of Engineering, Wuhan 430033, China; 2 Faculty of Information Engineering and Automation, Kunming University of Science and Technology, Kunming 650500, China; 3 Aerospace Information Research Institute, Chinese Academy of Sciences, Beijing 100190, China; 4 School of Electronic, Electrical and Communication Engineering, University of Chinese Academy of Sciences, Beijing 100049, China; 5 Personalized Management of Chronic Respiratory Disease, Chinese Academy of Medical Sciences, Beijing 100190, China

**Keywords:** blood pressure, carotid artery, diameter waveforms, pulse wave analysis, ultrasound

## Abstract

Blood pressure estimation through pulse wave analysis (PWA) aims to establish the relationship between features of pulse waveforms and blood pressure. This study is the first to investigate the connection between features of carotid artery diameter waveforms and variations in blood pressure, as well as to develop a blood pressure estimation model based on these features. A dataset was constructed from 14 subjects, with data collected across various physiological states and time points. For each subject, carotid artery diameter waveforms were measured using ultrasound, while synchronous blood pressure data were recorded with a reference device. A total of 52 morphological features were extracted from the diameter waveforms and their first and second derivatives. The influence of different models and feature combinations on blood pressure estimation was analyzed using various machine learning approaches. Ultimately, optimal models were developed for each subject to dynamic blood pressure fluctuations. On independent test data where blood pressure fluctuations exceeded 25 mmHg, the mean absolute error (MAE) of the estimates was 3.3 ± 4.1 mmHg. Even after a period of two days or more, the models remained effective, yielding a MAE of 4.2 ± 5.3 mmHg.

## 1. Introduction

Blood pressure is a critical indicator for assessing cardiovascular health and diagnosing cardiovascular diseases. Maintaining normal blood pressure levels is essential for the proper functioning of the body and for preventing cardiovascular and cerebrovascular diseases [1,2,3]. By continuously monitoring and managing blood pressure, individuals can better understand their health status and take preventive measures to slow or halt the progression of potential cardiovascular issues.

The cuff-based Korotkoff sound method and oscillometric method is widely used for blood pressure measurement. These techniques require inflating a cuff to compress the artery, followed by recording oscillatory pressure waveforms either through auscultation or pressure sensors to derive systolic and diastolic pressures. However, this method demands professional healthcare personnel to perform the measurement according to specific procedures. The results can also be influenced by factors such as cuff size, tightness, and the skill of the operator, potentially leading to inaccurate readings [4]. Additionally, this technique only provides intermittent blood pressure readings and does not allow for continuous monitoring.

To overcome the limitations of traditional blood pressure measurement methods, researchers have been exploring more portable and accurate alternatives. Notable among these are the pulse transit time (PTT, or pulse wave velocity, PWV) method and PWA method. Both approaches utilize physiological signal waveforms, do not rely on cuffs, and have the potential for continuous monitoring. The PTT method measures pulse wave signals using two or more pulse sensors and evaluates the time difference between these signals to estimate blood pressure through PWV [5]. A comprehensive review of PTT-based blood pressure measurement can be found in Mukkamala et al. [6]. PTT-based models typically disregard variations in arterial diameter, wall thickness, and elastic modulus, representing a significant simplification of the physiological system [7]. Consequently, PTT alone cannot fully capture dynamic blood pressure changes, since it is influenced by multiple cardiovascular factors beyond arterial pressure, which in turn also affect PTT.

Compared to the PTT method, the PWA technique requires only a single sensor to capture the pulse waveform, making it a more practical and accessible approach. Additionally, the waveform may contain various features that correlate with the state of the cardiovascular system. The PWA technique extracts morphological features from arterial waveforms and maps them to blood pressure using predictive models. The pulsatile blood flow generated by the heart’s cyclical pumping propagates through the arterial system, forming a characteristic blood pressure waveform. The maximum and minimum pressures in this waveform correspond to systolic and diastolic blood pressure (SBP and DBP), respectively. At points of arterial bifurcation, terminal organs, or vascular discontinuities, forward-traveling pressure waves encounter changes in transmission impedance, generating reflected waves. As a result, the blood pressure waveform is the superposition of a forward wave originating from the aorta and a reflected wave from peripheral sites. Blood pressure is influenced by both cardiac output (the source of blood flow) and total peripheral resistance (the properties of the arterial pathway). Changes in either cardiac output or peripheral resistance manifest as alterations in the morphology of the blood pressure waveform [8]. For instance, as arterial stiffness increases with age, PWV accelerates, and the intensity of the reflected wave grows. This causes the reflected wave to return earlier, during late systole, elevating SBP. This effect is visible in the waveform as an increased augmentation index (AIx) [9] and altered timing of the reflected wave. After physical exercise, increased heart rate and contractility lead to elevated blood pressure, observable in the waveform as a shorter systolic upstroke. PWA-based blood pressure estimation aims to model the relationship between pulse wave morphology and blood pressure.

Current studies on blood pressure measurement via PWA rely primarily on photoplethysmography (PPG) [10,11,12]. PPG employs light-emitting diodes and photodetectors to estimate blood volume changes in subcutaneous arterioles, generating a pulsatile signal that correlates with blood pressure waveforms. However, the arterial beds monitored by PPG exhibit viscoelastic properties, leading to a nonlinear relationship between the PPG-derived volume waveform and the actual blood pressure waveform. In effect, the PPG signal can be regarded as a low-pass-filtered representation of the arterial pressure waveform at the measurement site, with the filter’s gain and cutoff frequency modulated by vascular smooth muscle activity [13]. Critically, such smooth muscle activity may vary independently of changes in blood pressure [13]. Additionally, factors including skin pigmentation, obesity, and external pressure on the sensor can alter the morphology of the PPG signal [14]. Collectively, these influences compromise the accuracy of PPG-based PWA methods for blood pressure estimation. Alternative pulse wave modalities have been explored for PWA-based blood pressure assessment, such as body-surface vibration signals and ballistocardiography (BCG) [15,16,17]. Nevertheless, compared to PPG, these signals offer a more indirect representation of the blood pressure waveform and exhibit greater deviation from the true hemodynamic morphology. They are also more vulnerable to confounding variables such as body mass, posture, measurement location, and sensor placement, posing additional challenges for implementing robust PWA-based blood pressure estimation with these modalities.

In contrast, ultrasound offers penetration capabilities that bypass the effects of tissue and small arteries, allowing for direct measurement of arterial diameter changes. Notably, in the case of large arteries such as the aorta (e.g., aorta, carotid artery), elasticity predominates while viscosity can be neglected, leading to a closer linear relationship between blood pressure waveforms and central arterial diameter waveforms. Furthermore, compared to peripheral small arterial beds, central arteries contain less smooth muscle, making them less susceptible to the effects of smooth muscle contraction and relaxation [6]. Several studies have shown that, when DBP and SBP are known, blood pressure waveforms can be derived by scaling arterial diameter (or cross-sectional area) waveforms using either exponential or linear equations [18,19]. Additionally, by incorporating arterial compliance, it is feasible to obtain pulse pressure (PP = SBP − DBP) and waveforms without calibration [20,21,22]. This suggests that the relationship between arterial diameter waveforms and blood pressure waveforms in central arteries is more direct. Thus, for constructing blood pressure estimation models, arterial diameter waveforms, particularly those of major arteries, may serve as superior signal sources compared to PPG (which characterizes the volume of small arterial). Recent studies have focused on miniaturizing ultrasound sensors for wearable applications [23,24,25,26], while others have aimed at compacting the entire ultrasound system [27]. This advancement allows ultrasound sensors to provide monitoring capabilities comparable to those of PPG sensors for daily use.

However, there is currently limited research focusing on the use of ultrasound arterial waveforms for PWA blood pressure estimation, and the underlying mechanisms, feasibility, and accuracy of this approach require further investigation. Nabeel P.M. et al. explored a blood pressure measurement method using ultrasound-derived arterial diameter waveforms [28]. They measured continuous carotid artery diameter waveforms in 20 individuals at rest and recorded a single blood pressure measurement. By segmenting the stable arterial diameter waveforms according to the cardiac cycle and dividing them into training and validation sets, they employed a deep learning model for training, which yielded satisfactory blood pressure estimates. In subsequent research, they investigated feature engineering methods using the same data [29]. However, both studies have limitations: each subject had only one static blood pressure measurement, failing to account for intra-subject blood pressure variations. Moreover, the continuous arterial diameter waveforms of each participant were simultaneously included in both the training and testing sets.

**Figure 1:**
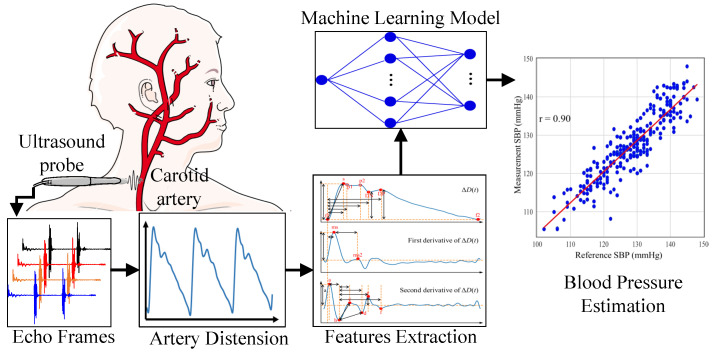
Technical roadmap of this work.

This work aims to investigate the relationship between arterial diameter waveform features and varying blood pressure during significant blood pressure fluctuations in subjects. It also seeks to develop personalized models for blood pressure assessment. To our knowledge, this is the first study to estimate blood pressure via PWA using arterial diameter waveforms under conditions of meaningful intra-subject blood pressure changes. Figure 1 illustrates the technical roadmap of this work.

## 2. Materials and Methods

This study utilized a custom-designed single-sensor A-Mode ultrasound system to acquire continuous echo data from the anterior and posterior walls of the carotid artery, enabling the extraction of continuous arterial diameter distension waveforms (ΔD(t)) for pulse wave analysis. Fourteen subjects participated in this research, undergoing multiple cold pressor tests and exercise sessions to induce variations in blood pressure while simultaneously measuring blood pressure and the corresponding changes in carotid artery diameter distension waveforms. For each subject, the training and testing datasets were derived from different experimental groups, ensuring the independence of the data and preventing any overlap. Features from the waveforms were extracted, and personalized models were developed using machine learning techniques, which were subsequently validated on independent test data.

### 2.1. Data Acquisition and Processing

The data acquisition hardware system is described in detail in our previous study [26]. In brief, it consists of a multi-channel ultrasound data transmission and reception system, while this work utilized only a single channel. High-voltage pulses excite the ultrasound transducer to emit ultrasound waves and collect echoes from tissues and arterial walls. The emitted pulse has a width of 100 ns and a high voltage of 60 V, with a pulse repetition frequency set at 200 Hz, resulting in a sampling rate of 200 Hz for the ΔD(t) waveforms, which is sufficient to capture the rich details of ΔD(t). The echoes are amplified by 48 dB and then digitized using an ADC with a sampling rate of 80 MHz. The acquired data is transmitted in real-time to a host computer via USB 3.0 interface for further processing. The ultrasound transducer used in this study has a central frequency of 5 MHz and a diameter of 5 mm. It is embedded within a 3D-printed structure to form a handheld probe, facilitating measurements by the operator.

**Figure 2:**
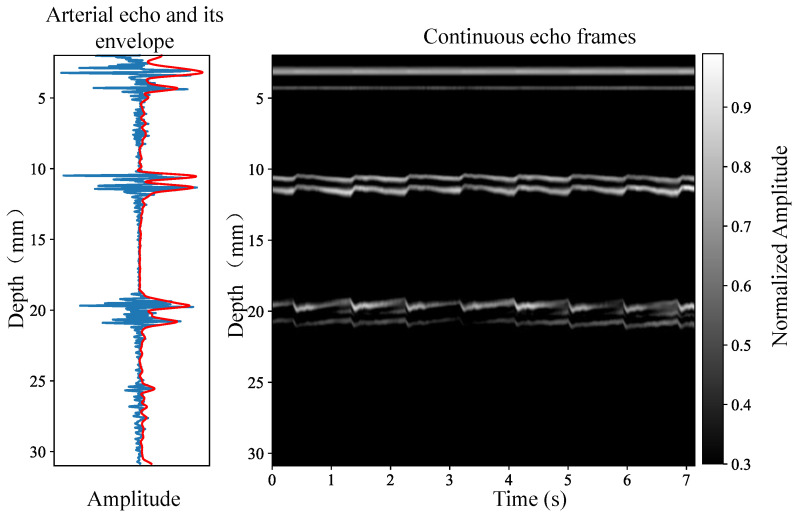
Single-frame arterial echo and continuous echo frames.

When the ultrasound beam is aligned with the artery, two distinct envelopes become apparent in the echo display due to the significantly higher echoes from the arterial walls compared to the surrounding tissues. The pulsation of the arterial walls, driven by changes in blood pressure, results in noticeable displacements in the echoes between frames. Figure 2 illustrates both the single-frame arterial echo and a schematic representation of continuous echo frames.

**Figure 3:**
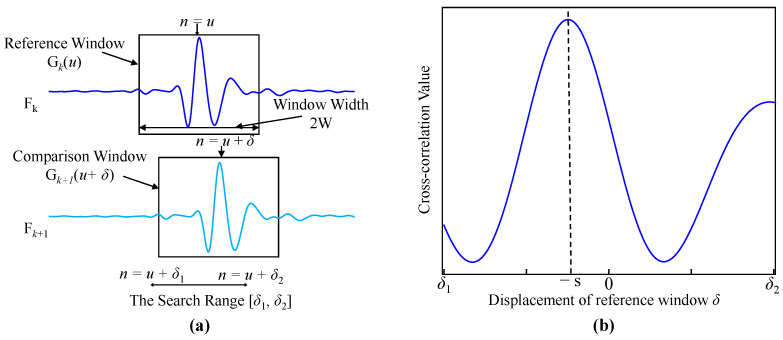
Principle of the CCA displacement estimator: (**a**) reference window and sliding comparison window; (**b**) variation of cross-correlation value with window sliding.

High-precision diameter distension waveforms are derived from consecutive echo frames using Echo-Tracking technology, the core of which is the Cross-Correlation Algorithm (CCA). First, the raw frames are filtered through a 4th-order bandpass filter with cutoff frequencies of 2 MHz and 8 MHz. Next, a region of interest (ROI) of 80 sampling points is selected, centered on the peak echo of the arterial wall. Given a speed of sound of 1540 m/s and a sampling rate of 80 MHz, 80 sampling points correspond to a range of 0.77 mm, covering the probable extent of the arterial wall. CCA is applied separately to the ROIs of the echoes from the proximal wall and the distal wall. The basic principle of CCA is to slide one signal relative to another and calculate the correlation coefficient at each shift to identify the position of maximum similarity between the two signals. Figure 3a illustrates this principle. For the *k*-th echo frame $F_{k}$, the approximate region containing a single arterial wall (proximal or distal) is denoted as $G_{k}$, which can be expressed as a function of the sample point *n*:

(1) $$G_{k}\left(n\right)=F_{k}\left(u-W+n\right),\;\;\;\;0<n\leq 2W\;$$

Here, $G_{k}$ is an echo segment (ROI) centered at u with a window length of 2W. Assuming the displacement of the arterial wall echo between frame k and frame *k* + 1 is $s_{k}$, the objective of cross-correlation displacement estimation is to compute $s_{k}$. By sliding the wall-echo window of frame *k* + 1 with a shift of δ sampling points, the cross-correlation C(δ) is calculated as

(2) $$C_{k}\left(\delta \right)=\;\sum_{n=u-W}^{u+W}G_{k}\;\left(n\right)\;G_{k+1}\left(n+\delta \right)\;\;\;\;\;\;\;\;\delta_{1}\leq \delta \leq \delta_{2}$$

where [δ1, δ2] defines the sliding range of the comparison window, which must cover physiologically plausible wall displacements and also account for potential probe motion during measurement. The value of δ that maximizes C(δ) gives an estimate of the negative of $s_{k}$:

(3) $${\hat{s}}_{k}=\underset{\delta }{-\arg\max}\;C_{k}\left(\delta \right)$$

Figure 3b shows the result of the cross-correlation computation. Finally, the cumulative frame-to-frame displacement from frame 0 to frame *N* yields the continuous displacement waveform for that wall:

(4) $$\Delta W_{k}=\sum_{k=0}^{N}{\hat{s}}_{k}$$

The continuous diameter distension waveform is then obtained as the difference between the displacement waveform of the proximal wall $\Delta W_{k\text{\_}p}$ and that of the distal wall $\Delta W_{k\text{\_}d}$:

(5) $$\Delta D_{k}=\Delta W_{k\text{\_}p}-\Delta W_{k\text{\_}d}$$

The accuracy of the CCA is not affected by the ultrasound frequency. After interpolating the raw echo signals, the estimation precision of arterial diameter distension can reach the micron level [30]. In this work, the raw ultrasound echoes were interpolated to 480 MHz before applying the CCA to obtain ΔD(t). Consequently, the theoretical resolution of ΔD(t) reaches 1.6 µm.

**Figure 4:**
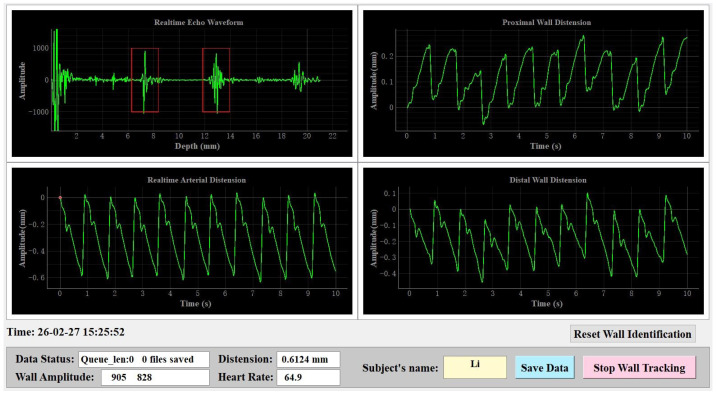
Screenshot of the software GUI for the data acquisition system.

The CCA algorithm is integrated into a host computer software with a graphical user interface (GUI, Figure 4) to process echo signals in real time. The software facilitates echo display, arterial wall identification, signal processing, and result visualization, while also providing data storage functionality. The software can automatically identify the arterial walls, allowing the operator to adjust the probe based on the echo patterns and identification results to ensure optimal positioning. Once the software detects the arterial walls, it automatically executes the CCA to assess the displacements of the anterior and posterior walls and to derive the arterial ΔD(t) waveform. Additionally, the operator can save the original echo frame data by clicking the “Save Data” button on the software interface. The saved data includes 12 s of raw echo data, timestamps. These timestamps are utilized for temporal alignment with blood pressure measurements from the reference device during subsequent processing.

### 2.2. Data Collection from Human Subjects

Fourteen healthy participants took part in the data collection experiment, including 11 males and 3 females, aged between 23 and 29 years (25.5 ± 2.0 years). Their Body Mass Index (BMI) ranged from 18.1 to 26.8 kg/m^2^ (22.2 ± 2.74 kg/m^2^). All participants were in good health and were instructed to refrain from smoking, consuming alcohol, and ingesting caffeine-containing foods for 24 h before the experiment to maintain relative cardiovascular stability. Measurements were obtained at the common carotid artery. This site provides a straight segment that is readily accessible via ultrasound and is classified as a central artery; it is minimally influenced by smooth muscle contraction, rendering it a suitable location for assessment. To elicit substantial blood pressure variations, participants underwent cold pressor and exercise interventions. Each experimental session consisted of a resting phase, a cold pressor phase, a recovery phase following the cold pressor test, and a post-exercise recovery phase.

To improve model reliability, it is necessary to collect as much data as possible from each subject. Hence, the selection of a reference blood pressure device is critical—it must support continuous blood pressure monitoring. This is particularly important during the cold pressor phase, where blood pressure changes rapidly. Conventional cuff-based sphygmomanometers require inflation and deflation, a process lasting over 30 s, which is insufficient for capturing rapid hemodynamic fluctuations. The Nexfin device, which utilizes the volume clamp method for continuous blood pressure measurement, is well-suited for this purpose and was employed as the primary reference instrument, especially during the resting and cold pressor phases.

In contrast, during the exercise phase, both ultrasound and reference blood pressure devices are significantly affected by motion artifacts, so only data from the recovery phase post-exercise are collected. It is important to note that the Nexfin device requires calibration during the first few minutes after being donned, a period during which blood pressure tends to return to baseline levels. Furthermore, even when the Nexfin device is worn during exercise, it has been observed that it does not accurately track the participant’s blood pressure post-exercise. For instance, while the heart rate may increase by over 60% following exercise, the Nexfin-measured blood pressure may only rise by approximately 10 mmHg from resting levels, which is inconsistent with expectations. During the recovery phase, the blood pressure recorded by the Nexfin device fluctuates instead of trending back towards baseline, with fluctuations reaching 10 mmHg. This discrepancy could be attributed to rapid heart rates or the contraction and relaxation of smooth muscle in the fingers, affecting the accuracy of Nexfin measurements. Similar findings have been reported by Raamat et al. using other constant volume clamp devices [31]. Conversely, the SunTech Oscar2 cuff blood pressure monitor accurately measures blood pressure following exercise. Therefore, the reference blood pressure device during the post-exercise recovery phase is the SunTech Oscar2 cuff blood pressure monitor.

Participants lay supine on the examination bed, with their heads resting on a pillow and turned to the right to adequately expose the left common carotid artery. A finger cuff from the Nexfin continuous blood pressure monitoring device was placed on the left middle finger, while the SunTech Oscar2 cuff blood pressure monitor was applied to the right arm. Five minutes after initiating measurements with the Nexfin device, internal calibration was completed, and stable continuous blood pressure readings were provided. To ensure consistency between the two reference devices, blood pressure was measured twice using the SunTech Oscar2 monitor before the ultrasound measurements, with a two-minute interval between readings. This consistency check was performed not to validate the accuracy of the reference devices themselves—as both are medically certified instruments with well-established reliability—but rather to identify any potential issues related to improper cuff or finger placement (e.g., incorrect sizing or positioning) that could affect measurement accuracy. If both measurements from the Oscar2 device differed from the Nexfin readings by no more than 5 mmHg, the subsequent measurement procedure continued. If not, the operator checked the proper placement of either the finger cuff or the arm cuff until the discrepancy between the two devices fell within the acceptable range of 5 mmHg. Following this, the ultrasound measurement procedure commenced. During data collection, the operator aligned the probe with the artery within the ultrasound field, while the software automatically identified the arterial wall and began calculating the diameter waveform. This information was displayed in real-time on GUI. The operator avoided applying excessive pressure with the probe to prevent deformation of the underlying carotid artery. When the operator pressed the save button, the software recorded 12 s of raw echo frame data. The saved ultrasound data included a local timestamp for alignment with the Nexfin blood pressure data.

**Figure 5:**
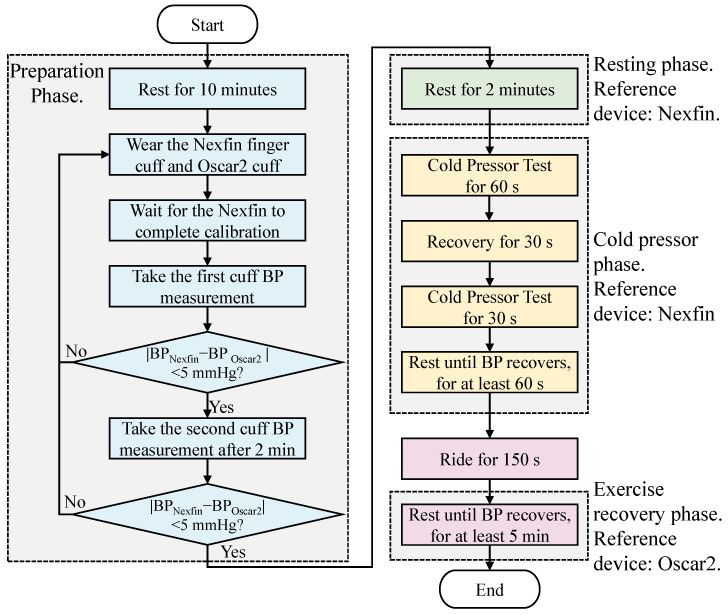
Data acquisition process of a data collection experiment for one group.

During the formal data collection phase, participants initially rested for 2 min, during which 2 to 3 segments of ultrasound data were collected. Subsequently, participants submerged their right hand in cold water at approximately 0–4 °C for data acquisition. The cold pressor phase consisted of 60 s of immersion, followed by a 30 s recovery period, another 30 s of immersion, and a recovery period lasting over 60 s until blood pressure returned to resting levels. If the maximum increase in blood pressure during the cold pressor test phase did not exceed 15 mmHg, the duration of immersion was extended. During this period, the operator collected approximately 8 to 14 segments of ultrasound data. Following the cold pressor phase, participants exercised on a stationary bike for 150 s, maintaining a power output of over 150 watts. After exercising, participants lay in the same position on the examination table for ultrasound and cuff blood pressure measurements. During testing, the SunTech Oscar2 blood pressure monitor performed a single measurement over approximately 30 to 40 s, capturing 12 s of ultrasound data during the intermittent deflation phase after inflation was completed. Measurements were taken at least 5 times per minute until blood pressure returned to baseline levels. Figure 5 illustrates the workflow of the data collection experiment.

**Figure 6:**
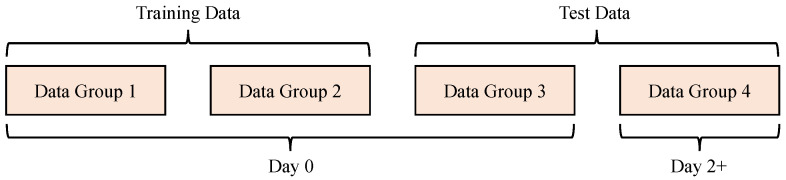
Division of training samples and test samples.

To ensure independence between the training and validation datasets and prevent any data overlap, each participant completed three data collection sessions within the same day. The first two sessions were used for model fitting, and the third session was reserved for testing model performance. Additionally, to evaluate the model’s long-term accuracy, follow-up sessions were conducted on a subset of participants: after two days (for 5 participants), seven days (for another 5 participants), or 21 days (for 4 participants). During these follow-ups, a complete fourth dataset was obtained. Each data collection session followed the protocol depicted in Figure 5, while Figure 6 illustrates the split between training and testing samples. The third and fourth datasets are designated as the short-term test set and the long-term test set, respectively.

### 2.3. Feature Extraction

**Figure 7:**
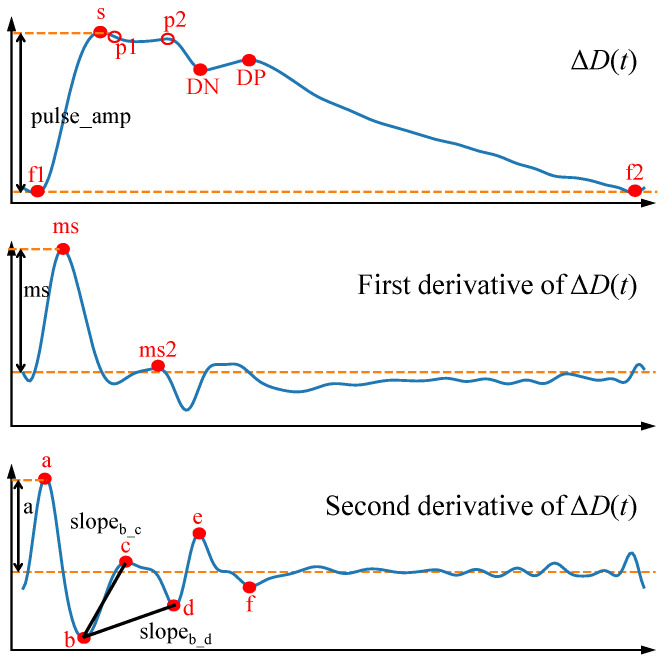
Definitions of fiducial points of ΔD(t); first and second derivative waveforms.

The continuous ΔD(t) waveform was then bandpass-filtered (fourth-order, 0.4–16 Hz) to remove baseline drift and high-frequency noise. A first-derivative Savitzky–Golay filter with a 5-point window was applied to the original ΔD(t) waveform to obtain its first and second derivatives. In accordance with the method introduced by Charlton et al. [32], fiducial reference points were identified from the ΔD(t) signal along with its first and second derivatives, as depicted in Figure 7. In this representation, $f_{1}$ and $f_{2}$ mark the onset and end of the cardiac cycle, respectively; s corresponds to the systolic peak; and $p_{1}$ and $p_{2}$ relate to early systolic pressure and the timing of reflected wave arrival. The dicrotic notch (DN) demarcates the transition between systole and diastole. Relevant extrema of the first and second derivative waveforms (velocity and acceleration) were also annotated. These fiducial points carry established physiological significance, and similar markers have been validated in previous studies as correlates of cardiovascular function and blood pressure variation. Comprehensive definitions for these points are provided by Charlton et al. [32].

A total of 52 features were extracted from these fiducial points. These comprise 25 features related to time intervals and ratios, 21 features related to relative amplitude, 2 features based on slopes (derived from amplitude and time ratios), and 4 area-based features. Their definitions are summarized in Table 1. In the table, “amp” refers to the amplitude difference between the peak and trough of the ΔD(t) waveform, while the prefix “amp” before a point designation indicates the amplitude at that specific fiducial point for ΔD(t) and its first and second derivatives.

### 2.4. Model Construction and Training

The intricate nature of the cardiovascular system poses significant challenges in developing a generalized model that can be effectively applied to all subjects, even with extensive training datasets. Previous studies utilizing population calibration strategies in PWA blood pressure measurement have often reported suboptimal accuracy [33,34,35]. Additionally, there is currently no publicly available dataset linking arterial diameter waveforms with blood pressure. As this research is the first to investigate the relationship between these waveforms and blood pressure variations, gathering sufficient data for a robust generalized model within a short timeframe proves difficult. Therefore, this study will focus on constructing a personalized model for each subject.

This work utilizes only the 52 features listed in Table 1 for training. It is noted that changes in DBP during exercise and cold pressor are less pronounced than those in SBP. Consequently, when both training and testing sets exhibit small numerical variations, the model may achieve low error on the test set, but it remains unclear whether this is due to the significant contribution of features or the limited variability of the data. Therefore, this study focuses the fitting objective solely on SBP.

Due to the small dataset size, larger models such as deep neural networks may be prone to overfitting, while simpler models can yield better performance and greater interpretability. Given this consideration, this study selects two nonlinear models (Random Forest, RF, and eXtreme Gradient Boosting, XGBoost) and one linear model (Multiple Linear Regression, MLR) as the primary machine learning models. RF enhances accuracy and generalization by constructing multiple decision trees and aggregating their estimates, making it particularly suitable for small datasets as it reduces overfitting risk through the introduction of randomness in samples and features [36]. XGBoost is an efficient gradient boosting framework that optimizes estimation performance by sequentially adding tree models, employing fine-tuned regularization to control overfitting, and performing well even on small datasetscite [37]. MLR, on the other hand, is a statistical method used to estimate the linear relationship between a dependent variable and multiple independent variables.

To compare the accuracy of the chosen models, this study employs a Mean Value Model (MVM) based on the training set, which uses the mean of the training data to estimate the test set. The MVM model serves as a benchmark to assess whether there are significant changes in blood pressure and to evaluate the contribution of the extracted features and employed models for blood pressure estimation.

## 3. Results

### 3.1. Dataset Statistics

**Figure 8:**
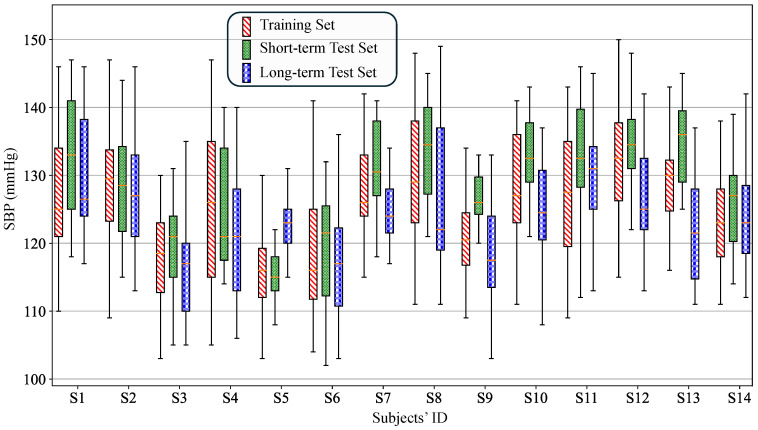
Boxplot of SBP distribution for 14 subjects.

In the constructed dataset, the amount of training data (Groups 1 and 2) per subject averaged 39.1 ± 8.3 entries, while the short-term test data (Group 3) averaged 18.9 ± 3.9 entries, and the long-term test data (Group 4) averaged 24.1 ± 6.3 entries. Regarding blood pressure distribution, the mean SBP for the training set, short-term test set, and long-term test set across all 14 subjects were 125.4 ± 10.0 mmHg, 128.0 ± 9.6 mmHg, and 124.2 ± 9.3 mmHg, respectively. For individualized calibration models, the blood pressure distribution of individual datasets is more critical than the overall SBP distribution. The range of SBP across the three datasets exceeded 25 mmHg. Using resting blood pressure as a baseline, the average SBP changes within subjects were 15.3 ± 7.7 mmHg, 13.7 ± 6.8 mmHg, and 13.2 ± 7.8 mmHg, respectively. These results indicate that the designed experiment successfully induced significant SBP variations in the subjects, with average changes exceeding 13 mmHg compared to resting levels across all three datasets. Figure 8 shows the box plots of SBP distribution for each subject across the three datasets.

### 3.2. Feature Ranking

With an average of 39.1 ± 8.3 data points per participant and a total of 52 features, the analysis focused on the top 20 features (approximately 50% of the average training dataset size) to mitigate the risk of overfitting for each subject-specific model. Significant variability in feature rankings was observed across individuals, reflecting inherent physiological differences; cardiovascular parameters naturally vary between subjects, and their response patterns during cold pressor tests and after exercise may also differ. To systematically evaluate the importance and consistency of features across datasets, a feature ranking and composite scoring method was implemented, as outlined below:

(1) Within-Subject Feature Ranking: For each participant, feature importance was independently assessed using the respective training set, resulting in a ranked list of all 52 features based on their contribution to model performance.

(2) Feature Selection and Scoring: From each subject’s ranking, the top 20 features were selected. A score was assigned to each selected feature according to its rank: the top-ranked feature received 20 points, the second 19 points, and so on, with the 20th-ranked feature receiving 1 point.

(3) Composite Scoring and Final Ranking: Scores for each feature were aggregated across all 14 subjects to compute a composite score. Features were then globally ranked based on these composite scores to determine their overall importance.

**Figure 9:**
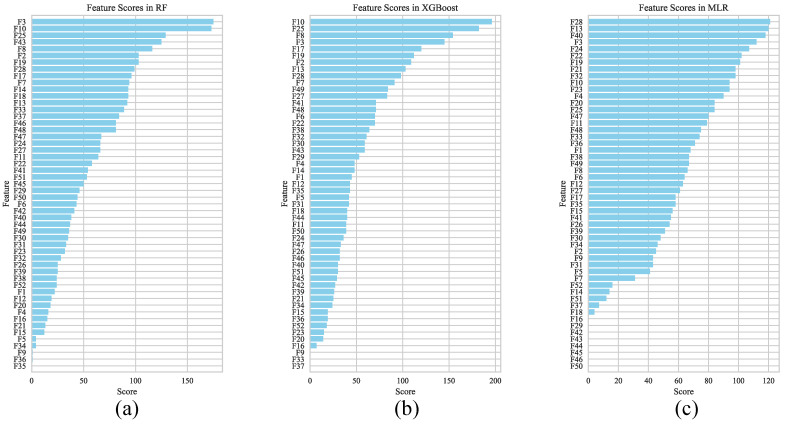
Feature score rankings of three models: (**a**) RF; (**b**) XGBoost; (**c**) MLR.

Figure 9 displays the final composite scores and rankings for the Random Forest (RF), XGBoost, and Multiple Linear Regression (MLR) models across the test sets of the 14 subjects. Feature importance rankings differed notably among the models, largely due to variations in their underlying principles, assumptions regarding data distribution and structure, and model complexity.

In the nonlinear models (RF and XGBoost), several features demonstrated high importance: F2 ($t_{b}$), F3 ($t_{c}$), F25 ($T_{b\text{\_}c}$), F8 ($t_{sys}$, systolic timing), F19 (prop_s, proportion of the ascending phase), and F10 ($t_{p1}$). Notably, all six are time-related features. F2, F3, and F25 are parameters associated with points *b* and *c* on the second derivative waveform, which have previously been shown to aid blood pressure estimation from photoplethysmography (PPG) signals [38]. F8 [39] and F19 [40] relate to cardiac contraction timing and velocity, reflecting cardiac output dynamics. F10 represents the time from point $p_{1}$ to the cycle onset, indicating the arrival time of the reflected wave and thus being associated with arterial stiffness. In contrast, within the linear MLR model, the top three ranked features were F28 (amp_p2), F13 ($t_{MS2}$), and F40 (d_div_a).

### 3.3. Blood Pressure Estimation Results Analysis

**Figure 10:**
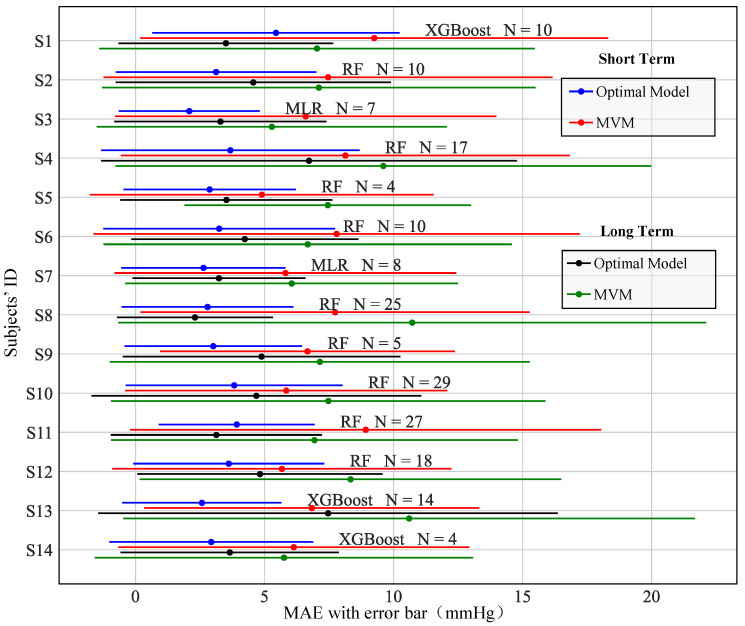
Errors of the best individual calibration models for the 14 subjects, wgere N denotes the number of features used by the optimal model.

In the context of individual calibration strategies, the best model and the number of features varied among different subjects. Figure 10 displays the MAE with standard deviation (SD) error bars for the best individual calibration for each subject across short-term and long-term test sets. To provide a clearer visual representation of the optimal model’s performance, the estimated errors from the mean value model (MVM) are also included. Among the 14 optimal individual calibration models, 9 were based on the RF model, 3 on the XGBoost model, and 2 on the MLR model. Given the significant changes in blood pressure within individuals and the fact that the training and test sets originated from different experimental groups, the MAEs for each subject’s best individual calibration model were 3.3 ± 4.1 mmHg for the short-term test set and 4.2 ± 5.3 mmHg for the long-term test set. These results demonstrate a significant improvement compared to the baseline MAEs of 7.0 ± 7.9 mmHg and 7.4 ± 8.9 mmHg from the MVM model.

**Figure 11:**
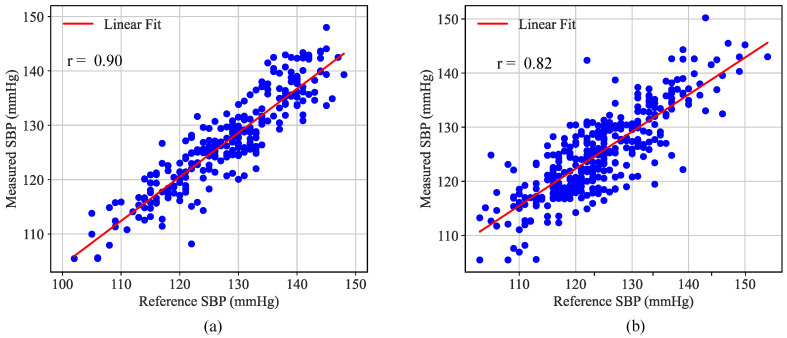
Correlation between the estimated and reference blood pressure values across the 14 participants for the (**a**) short-term and (**b**) long-term test sets.

Figure 11 shows the correlation plots for all measurement points when using each subject’s best individual calibration model on their respective short-term and long-term test sets. The correlation coefficients between estimated values and true blood pressure are r = 0.90 for the short-term test set and r = 0.82 for the long-term test set. The results demonstrate that the individualized calibration models retained a high level of accuracy during blood pressure fluctuations; even though model performance exhibited a moderate decline beyond two days, overall, these models remained effective. This performance decline is primarily attributable to natural fluctuations in the participants’ physiological states over time, including variations in cardiac output, arterial stiffness, and vascular reactivity due to factors such as circadian rhythms, emotional states, and metabolic changes. These physiological drifts can alter the underlying relationship between arterial diameter waveform features and blood pressure, leading to a moderate reduction in model accuracy. Nevertheless, given the challenging nature of long-term physiological monitoring and the absence of any model recalibration over intervals ranging from two days to more than one week, the MAE of 4.2 ± 5.3 mmHg and the correlation coefficient of r = 0.82 demonstrate the robustness and reliability of the proposed method for tracking blood pressure changes in real-world scenarios. In future work, we plan to address this drift by incorporating periodic model updating strategies, such as intermittent recalibration with reference blood pressure measurements or online learning mechanisms, to adapt to gradual physiological changes and further enhance long-term stability.

**Figure 12:**
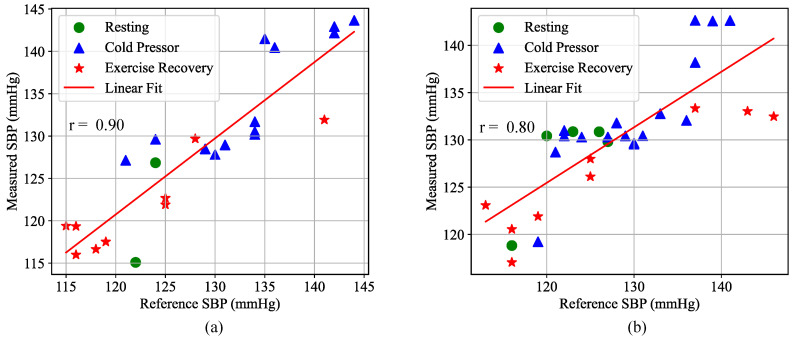
Performance of participant S2’s model on the dataset: (**a**) short-term test sets; (**b**) long-term test sets.

Figure 12 compares the estimated blood pressure and true blood pressure from the best individualized calibration model (RF, with 10 features) for participant S2 across short-term and long-term test datasets. Different scatter point types represent various physiological states. This participant exhibited a wide range of blood pressure distributions in both test sets, with variations exceeding 30 mmHg, indicating significant blood pressure changes during the data collection experiment. The best model for this participant effectively tracked the changes in true values, with estimated errors of MAE = 3.1 ± 3.9 mmHg for the short-term test sets and MAE = 4.6 ± 5.3 mmHg for the long-term test sets. The correlation between estimated values and true blood pressure was r = 0.90 and r = 0.80, respectively.

These results suggest that features extracted from arterial diameter waveforms effectively reflect blood pressure changes, and models built using these features can accurately estimate fluctuating blood pressure, remaining effective even after 2 days or longer.

## 4. Discussion

In this study, we collected significantly varying blood pressure data and synchronized ultrasound arterial diameter distension waveform data from 14 participants across three physiological states: resting, cold pressor, and post-exercise. The aim was to analyze the relationship between arterial morphological features and fluctuating blood pressure, leading to the development of a blood pressure estimation model. The model demonstrated satisfactory estimation accuracy on an independent test set, remaining effective even after 2 days or longer. To our knowledge, this is the first work to utilize arterial diameter waveforms for PWA blood pressure measurement under induced blood pressure changes. This study demonstrates the strong potential of ultrasound-measured arterial morphological waveforms for blood pressure estimation. Although the current prototype requires manual operation, advances in microelectronics could enable the integration of this technique with wearable ultrasound systems, ultimately paving the way for wearable applications and continuous blood pressure monitoring.

In previous studies, a representative technique for measuring blood pressure via ultrasonic arterial morphology relies on an empirically derived linear or exponential relationship between the pressure exerted on the artery and its cross-sectional area, which has been applied to miniaturized flexible ultrasound arrays [23]. This technique necessitates the use of an external blood pressure monitor to measure SBP and DBP to determine the arterial stiffness coefficient, and it lacks continuous calibration capability when arterial elasticity changes. Another approach uses local pulse wave velocity (PWV) to quantify arterial compliance, allowing for tracking of the pulse pressure (SBP − DBP) waveform [20,21,22]; we have previously validated this method using conventional ultrasound probes [22] and wearable flexible ultrasound sensors [41]. However, this method only provides pulse pressure, leaving absolute pressure levels unmeasured. This work addresses this gap by enabling absolute pressure measurement, thus providing a complete picture of blood pressure and its waveform in conjunction with previous findings.

This study did not incorporate absolute arterial diameter as an input feature, despite its potential physiological relevance. Accurately measuring arterial diameter using a single ultrasound transducer—without the aid of imaging—presents significant technical challenges. The ultrasound field intensity propagates axially outward rather than forming a precise linear beam, which means that received echoes are not exclusively derived from the direction of the arterial diameter. Although the use of smaller transducers could be considered, this approach introduces additional measurement complexity and may impair the reliable detection of arterial echoes, especially during periods of rapid hemodynamic change such as acute blood pressure fluctuations. Additionally, precisely identifying the intima–lumen interface from echoes poses significant challenges for accurately measuring absolute arterial diameter. Conversely, the CCA can obtain high-precision relative changes in arterial diameter through the accurate relative displacement of arterial walls, allowing for reliable capture of relative changes. The amplitude-related features used in this work are all relative quantities, ensuring their accuracy. Future research will consider utilizing closely arranged array ultrasound sensors to acquire precise absolute arterial diameters and explore their role in the PWA model.

The PWA blood pressure measurement method relies on data-driven models, and appropriate data partitioning is crucial for obtaining reliable results. Some previous studies have unreasonably split continuous or adjacent segments of data into training and testing sets, leading to artificially high but unrealistic predictive accuracy. As noted by Costa et al. in their review on PWA blood pressure measurement, the model may be “*learning to identify patients and not predict BP*” [42]. This highlights that, under such experimental settings, the model’s task becomes identifying subjects rather than estimating blood pressure [42]. This occurs because an individual’s pulse wave and blood pressure data do not exhibit significant changes within short timeframes, making the two segments of similar data highly analogous, with nearly identical corresponding blood pressures. In these circumstances, while the model may perform well on the original dataset due to overfitting, it may fail to produce ideal results on new data from the same subject. In practical applications, it is unlikely to encounter a scenario where a model is both trained and tested on a single segment of stable or continuous data. Mukkamala et al. emphasized the importance of inducing internal blood pressure variability in PWA blood pressure estimation methods that utilize the same subject’s data for both training and validation [43]. Considering these issues, this work has been carefully designed to closely reflect real-world situations and avoid overfitting. The collected data exhibited a sufficiently large range of blood pressure variations and accounted for different physiological states. Significant blood pressure variations not only better reflect the fluctuations observed in real-world scenarios but also help prevent the model from overfitting to individual subject data. These considerations make our model more aligned with practical situations. Both exercise and cold pressor stimuli were employed to alter the two main factors influencing blood pressure: cardiac output and total peripheral resistance. Exercise primarily increases blood pressure by enhancing cardiac output, whereas cold pressor primarily raises blood pressure by increasing total peripheral vascular resistance [44]. In data partitioning, training and testing datasets are independent of each other, with the testing set (groups 3 and 4) coming from different experimental groups than the training set (groups 1 and 2). Notably, the collection times for the testing data in group 4 and the training data were separated by more than two days, ensuring the rigor of the results.

Overall, this work demonstrates the feasibility of using arterial morphological features to measure changes in blood pressure. In future studies, we aim to collect data from a broader population and under a wider range of physiological states to investigate the deeper relationships between arterial morphology and blood pressure. This will enable us to explore universal models based on population calibration strategies, moving this technology closer to practical application. Furthermore, by integrating this method with emerging wearable flexible ultrasound arrays, we aim to transition this technology from controlled laboratory settings to continuous, real-world blood pressure monitoring.

## 5. Conclusions

This work represents the first study of the relationship between arterial morphological waveform features and fluctuating blood pressure, leading to the development of a blood pressure estimation model based on these morphological characteristics. We constructed a dataset comprising 14 participants, collecting arterial diameter distension waveforms and synchronized blood pressure data during resting, cold pressor, and post-exercise states to ensure significant blood pressure variations. By extracting 52 morphological features from the arterial diameter distension waveforms and their first and second derivatives, we analyzed the impact of different feature combinations and quantities on blood pressure estimation using RF, XGBoost, and MLR models. Optimal models were developed for each participant to estimate changing blood pressure levels. On an independent test dataset from the same day, where blood pressure changes were significant (with the reference model MVM showing a MAE of 7.0 ± 7.9 mmHg), our constructed model achieved a MAE of 3.3 ± 4.1 mmHg, demonstrating high measurement accuracy. Even with data collected after two days or longer, where the reference model’s error was MAE = 7.4 ± 8.9 mmHg, our model maintained an estimation error of MAE = 4.2 ± 5.3 mmHg, indicating its continued effectiveness. These findings suggest that features selected from arterial morphological waveforms and the models built upon them can effectively estimate changes in blood pressure. This work may contribute to the future development of robust PWA blood pressure measurement models based on arterial morphological characteristics.

## Figures and Tables

**Table 1:** Definitions of features derived from fiducial points of the ΔD(t) waveform and its derivatives.

ID	Feature Name	Feature Definition
Time-based (F1–F6)	t_a, t_b, t_c, t_d, t_e, t_f	Time interval from the fiducial point to the cycle start point $f_{1}$.
Time-based (F7–F13)	$t_{sys}$, $t_{DN}$, $t_{DP}$, $t_{p1}$, $t_{p2pk}$, $t_{MS}$, $t_{MS2}$	Time interval from the fiducial point to the cycle start point $f_{1}$.
Slope (F14)	STT	$\mathrm{amp}/t_{sys}$ (amplitude-to-systolic time ratio).
Cycle Duration (F15)	T	$t_{f2}-t_{f1}$ (total cardiac cycle duration).
Heart Rate (F16)	IHR	60/T (instantaneous heart rate).
Systolic Timing (F17)	ΔT	$t_{DN}-t_{sys}$ (time from systolic peak to dicrotic notch).
Systolic Index (F18)	SI	$t_{sys}/\mathrm{height}$ (systolic time normalized by height).
Systolic Proportion (F19)	prop_s	$t_{sys}/T$ (systolic phase proportion).
Diastolic Duration (F20)	$T_{dia}$	$t_{f2}-t_{DN}$ (diastolic interval).
Diastolic Ratio (F21)	$T_{ratio}$	$t_{DN}/T_{dia}$ (systolic-to-diastolic time ratio).
Timing Proportion (F22)	prop_ΔT	ΔT/T (normalized systolic decline time).
Pulse Timing 1 (F23)	$T_{p1\text{\_}dia}$	$t_{DP}-t_{p1}$ (time from $p_{1}$ to diastolic peak).
Pulse Timing 2 (F24)	$T_{p2pk\text{\_}dia}$	$t_{DP}-t_{p2pk}$ (time from $p_{2pk}$ to diastolic peak).
Interval 1 (F25)	$T_{b\text{\_}c}$	$t_{c}-t_{b}$ (time between points *b* and *c*).
Interval 2 (F26)	$T_{b\text{\_}d}$	$t_{d}-t_{b}$ (time between points *b* and *d*).
Amplitude Ratio 1 (F27)	amp_p1	amp_p1/amp (amplitude at $p_{1}$ relative to pulse amplitude).
Amplitude Ratio 2 (F28)	amp_p2	amp_p2/amp (amplitude at $p_{2}$ relative to pulse amplitude).
Augmentation Index (F29)	AIx	100×(amp_p2pk−amp_p1in)/amp.
Reflection Index 1 (F30)	RI_p1	amp_dia/amp_p1.
Reflection Index 2 (F31)	RI_p2	amp_dia/amp_p2.
Amplitude Ratio 3 (F32)	ratio_p2_p1	amp_p2/amp_p1.
Reflection Index (F33)	RI	amp_DP/amp.
Area 1 (F34)	A1	Integral under the curve left of the dicrotic notch (DN).
Area 2 (F35)	A2	Integral under the curve right of the dicrotic notch (DN).
Area Ratio (F36)	IPA	A2/A1 (inflection point area ratio).
Slope Metric (F37)	ms_div_am	ms/amp.
Amplitude Ratios (F38–F41)	b_div_a, c_div_a, d_div_a, e_div_a	amp_b/amp_a, amp_c/amp_a, amp_d/amp_a, amp_e/amp_a.
Normalized Amplitudes (F42–F46)	a_div_amp, b_div_amp, c_div_amp, d_div_amp, e_div_amp	amp_a/amp, amp_b/amp, amp_c/amp, amp_d/amp, amp_e/amp.
Aging Index (F47)	AGI	(amp_b−amp_c−amp_d−amp_e)/amp_a.
Aging Index Variant 1 (F48)	AGI_inf	(amp_b−amp_e)/amp_a.
Aging Index Variant 2 (F49)	AGI_mod	(amp_b−amp_c−amp_d)/amp_a.
Slope 1 (F50)	slope_b_c	(amp_d−amp_b)/(t_c−t_d).
Slope 2 (F51)	slope_b_d	(amp_d−amp_b)/(t_c−t_d).
Composite Index (F52)	IPAD	IPA + d_div_a.

## Data Availability

The unique dataset supporting this study was meticulously collected by the authors and represents a significant investment of resources. It is currently being utilized in several ongoing and planned investigations by our research team. To foster collaboration while protecting this investment, the data can be made available to qualified researchers under a formal data use or collaboration agreement. Interested parties are encouraged to contact the corresponding authors to discuss potential access.

## Abbreviations

The following abbreviations are used in this manuscript:

PWA	Pulse Wave Analysis
PTT	Pulse Transit Time
PWV	Pulse Wave Velocity
DBP	Diastolic blood pressure
SBP	Systolic Blood Pressure
PP	Pulse Pressure
PPG	Photoplethysmography
BCG	Ballistocardiography
ΔD(t)	Arterial Diameter Distension Waveforms
CCA	Cross-Correlation Algorithm
GUI	Graphical User Interface
RF	Random Forest
XGBoost	eXtreme Gradient Boosting
MLR	Multiple Linear Regression
MVM	Mean Value Model
MAE	Mean Absolute Error
